# Supplementary material for: Modeling glioblastoma heterogeneity as a dynamic network of cell states
Source: Mol Syst Biol. 2021 Sep 16;17(9):e10105. doi: 10.15252/msb.202010105 (PMC8444284; doi:10.15252/msb.202010105)
Supplement: Supplementary file 6 — Source Data for Figure 5 [file MSB-17-e10105-s004.zip › Figure5A_sourcedata/GSEA_3071/nosigdb_stateVI.GseaPreranked.1622795169056/SEGERMAN_MULTITHERAPY_RESISTANCE_FIG5A_PMID_27974212.html]

Details for gene set SEGERMAN\_MULTITHERAPY\_RESISTANCE\_FIG5A\_PMID\_27974212[GSEA]

|  || Dataset | state63071 |
| Phenotype | NoPhenotypeAvailable |
| Upregulated in class | na\_pos |
| GeneSet | SEGERMAN\_MULTITHERAPY\_RESISTANCE\_FIG5A\_PMID\_27974212 |
| Enrichment Score (ES) | 0.4670435 |
| Normalized Enrichment Score (NES) | 1.9722217 |
| Nominal p-value | 0.003508772 |
| FDR q-value | 0.007176717 |
| FWER p-Value | 0.058 |
Table: GSEA Results Summary

  

Fig 1: Enrichment plot: SEGERMAN\_MULTITHERAPY\_RESISTANCE\_FIG5A\_PMID\_27974212      
 Profile of the Running ES Score & Positions of GeneSet Members on the Rank Ordered List

  

| PROBE | GENE SYMBOL | GENE\_TITLE | RANK IN GENE LIST | RANK METRIC SCORE | RUNNING ES | CORE ENRICHMENT || 1 | DAB2 |  |  | 78 | 0.788 | 0.0744 | Yes |
| 2 | AHNAK |  |  | 139 | 0.680 | 0.1407 | Yes |
| 3 | SERPINB1 |  |  | 184 | 0.618 | 0.2038 | Yes |
| 4 | QSOX1 |  |  | 187 | 0.609 | 0.2775 | Yes |
| 5 | EDIL3 |  |  | 219 | 0.589 | 0.3407 | Yes |
| 6 | MSRB3 |  |  | 242 | 0.570 | 0.4040 | Yes |
| 7 | COPZ2 |  |  | 457 | 0.469 | 0.4020 | Yes |
| 8 | SLC25A43 |  |  | 604 | 0.423 | 0.4133 | Yes |
| 9 | MVP |  |  | 642 | 0.413 | 0.4534 | Yes |
| 10 | LOXL1 |  |  | 879 | 0.364 | 0.4325 | Yes |
| 11 | SHC1 |  |  | 913 | 0.358 | 0.4670 | Yes |
| 12 | CLMP |  |  | 1148 | 0.323 | 0.4418 | No |
| 13 | FN1 |  |  | 1654 | 0.264 | 0.3345 | No |
| 14 | EPHX1 |  |  | 2327 | -0.339 | 0.1903 | No |
| 15 | LAMA5 |  |  | 2447 | -0.366 | 0.2020 | No |
| 16 | EMILIN1 |  |  | 2936 | -0.507 | 0.1290 | No |
| 17 | PPIC |  |  | 2982 | -0.530 | 0.1811 | No |
Table: GSEA details [plain text format]

  

Fig 2: SEGERMAN\_MULTITHERAPY\_RESISTANCE\_FIG5A\_PMID\_27974212: Random ES distribution      
 Gene set null distribution of ES for **SEGERMAN\_MULTITHERAPY\_RESISTANCE\_FIG5A\_PMID\_27974212**
